# The estimation and interpretation of ordered logit models for assessing the factors connected with the productivity of Holstein–Friesian dairy cows in Egypt

**DOI:** 10.1007/s11250-022-03329-x

**Published:** 2022-10-15

**Authors:** Sherif A. Moawed, Ayman H. Abd El-Aziz

**Affiliations:** 1 Department of Animal Wealth Development (Biostatistics Division), Faculty of Veterinary Medicine, Suez Canal University, Ismailia, 41522 Egypt; 2 Animal Husbandry and Animal Wealth Development Department, Faculty of Veterinary Medicine, Damanhour University, Damanhour, 22511 Egypt

**Keywords:** Ordered logit models, Odds ratio, Proportional odds, Predicted probability, Holstein–Friesian dairy cows, Milk production

## Abstract

The incorporation of novel technologies such as artificial intelligence, data mining, and advanced statistical methodologies have received wide responses from researchers. This study was designed to model the factors impacting the actual milk yield of Holstein–Friesian cows using the proportional odds ordered logit model (OLM). A total of 8300 lactation records were collected for cows calved between 2005 and 2019. The actual milk yield, the outcome variable, was categorized into three levels: low (< 4500 kg), medium (4500–7500 kg), and high (> 7500 kg). The studied predictor variables were age at first calving (AFC), lactation order (LO), days open (DO), lactation period (LP), peak milk yield (PMY), and dry period (DP). The proportionality assumption of odds using the logit link function was verified for the current datasets. The goodness-of-fit measures revealed the suitability of the ordered logit models to datasets structure. Results showed that cows with younger ages at first calving produce two times higher milk quantities. Also, longer days open were associated with higher milk yield. The highest amount of milk yield was denoted by higher lactation periods (> 250 days). The peak yield per kg was significantly related to the actual yield (*P* < 0.05). Moreover, shorter dry periods showed about 1.5 times higher milk yield. The greatest yield was observed in the 2nd and 4th parities, with an odds ratio (OR) equal to 1.75, on average. In conclusion, OLM can be used for analyzing dairy cows’ data, denoting fruitful information as compared to the other classical regression models. In addition, the current study showed the possibility and applicability of OLM in understanding and analyzing livestock datasets suited for planning effective breeding programs.

## Introduction

The main objective of dairy cattle investment is to increase the amount of milk yield and to gain one calf every year with fixed and optimum intervals. Milk yield as a quantitative trait could be affected by environmental effects along with the genotypes of animals (Topal et al. 2010; Ayalew et al. 2015). The genetic improvement of such a trait in dairy populations requires accurate information and precise estimates of genetic parameters. However, it is necessary for the estimation of genetic parameters to acknowledge the incorporation of different environmental factors influencing the studied traits (Javed et al. 2007; Kuthu et al. 2007). Hence, genetic amelioration of dairy cow breeds to enhance milk yield as well as to identify the various factors influencing milk production should be accurately evaluated and specified (Topal et al. 2010). Moreover, acknowledging the environmental and genetic effects could be handled using various linear and nonlinear models and machine learning algorithms.

The major tricks and considerations that should be taken into account in statistical modeling are the data structure, assumptions behind the proposed model, as well as the types of both response and explanatory variables investigated (Christensen 2019; Oehm et al. 2022). Regression-based modeling is generally used to analyze the causal and functional relationship between a dependent response variable and one or more explanatory variables (Topal et al. 2010; Vittinghoff et al. 2012). In the ordinary least squares regression, the dependent variable should be normally distributed and fit a scale level of measurement. If the dependent variable is categorical with multiple levels, incorporating an ordinary linear regression model does not recommend due to violation of vital assumptions on the response variable, such as normality and linearity. In such cases, the use of other regression-based models could be beneficial for analyzing categorical outcomes, denoting more accurate and valid estimates (Akkus et al. 2019).

Logistic regression methods instead can be utilized to analyze and predict the categorical response variable on the basis of one or more explanatory variables (Adeleke and Adepoju 2010; Vittinghoff et al. 2012; Abd-El Hamed and Kamel 2021). Namely, the logistic regression models may be binary, ordinal, or polynomial, according to the number and nature of categories of the dependent variables. The main concept of all these logistic regression methods is to transform the given dependent outcome into an exponent function in the explanatory variables (Dayton 1992). In particular, when we are dealing with a categorical outcome with an ordered structure, an ordinal logistic regression method is strongly recommended over the other regression-based models to deem the ordered nature of the concerned variable (O’Connell 2011). Historically, ordinal logistic regression models have originally been suggested by Walker and Duncan (1967) and then named as proportional odds models, as proposed by previous authors (McCullagh 1980; Ananth and Kleinbaum 1997; Agresti 2007; Hosmer and Lemeshow 2000). In practice, the assumption of proportionality is rare to be verified (Agga and Scott 2015). In such cases, an unconstrained ordered logit model (a type of generalized ordered logistic regression) or partial proportional odds model can be applied to compensate for the proportionality assumption (Williams 2006; O’Connell 2011).

In the literature, although there is a number of studies using the binary logistic regression investigations in the area of milk yield predictions of Holstein–Friesian cows, a study using ordered logit models (OLM) for assessing milk yield calculations in dairy cattle is not existed in this extent, particularly in terms of estimation and interpretation. Therefore, we hypothesize that the introduction of OLM for assessing factors influencing milk production in dairy cows along with the odds ratio computations and interpretations would be a good reference for many researchers in the area of statistical modeling because the OLM is a flexible model and can be refitted using new variables for upcoming research. Also, the OLM is advantageous as it can provide estimated probabilities concerned with the milk yield. Unlike the other past regression-based models applied in the area, the estimated probabilities of milk yield will be gained through the estimated model with regard to the explanatory variables. In addition, the significance tests of the different levels of such explanatory variables will also be denoted so that required enhancements on the determinants influencing milk yield connected with those levels can be achieved.

There are various studies about OLM applications in dairy cattle investigations. In some of the previous studies, Vergara et al. (2014) used OLM to identify risk factors for postpartum problems in dairy cattle. Afonso et al. (2020) and Oehm et al. (2022) applied OLM to predict lameness in dairy cows. Ouweltjes et al. (2021) used OLM to predict the lifetime resilience of dairy cattle. O’Connor et al. (2020) studied the risk factors that impacted mobility scores in pasture-based dairy cattle using OLM. Furthermore, ordered logit models have been included in the literature, mostly for medical and epidemiological studies. Among these studies, Dong (2007) used the ordered logit models for predicting self-efficacy (an ordinal response) in inspecting different cancers of the colon and rectum. Adepoju and Adegbite (2009) used ordered logistic regressions to assess the correlations between staff categories as the response variable and each of gender, educational level, indigenous status, previous experience, and age as predictors. Adeleke and Adepoju (2010) conducted an inference study for modeling a categorical variable (pregnancy status) using ordered logit models.

However, up-to-date, the number of studies that have incorporated OLM and its algorithms in predicting the milk yield of dairy cattle is still limited. Moreover, the majority of investigations that have assessed factors regarded milk yield in dairy cows did not acknowledge the ordered nature of milk production trait being driven as a categorical variable. For example, Doğan and Özdamar (2003) and Berry et al. (2007) investigated the factors that impacted milk yield in Holstein Frisian cows using classical linear and nonlinear regression models. Other research carried out by previous authors (Akçay et al. 2007; Koçak et al. 2007; Tilki et al. 2008; Aksakal and Bayram 2009; Petrovic et al. 2009; Atashi et al. 2021; Vrhel et al. 2021) reported the effects of calving season, gender, type of birth, and number of births on birth weight of Holstein dairy cattle using analysis of variance procedures and the ordinary multiple regression models. Additionally, in recent studies by Valchev et al. (2020), Van Eetvelde et al. (2020), and Temesgen et al. (2022), the factors affecting milk yield were identified using regression-based models, but without handing the ordered nature of milk yield trait. In general, the previous investigations still entail a loss of information and accuracy in terms of providing comparable interpretations about milk yield determinants and their levels. Therefore, this study was designed to estimate and interpret the ordered logit models for acknowledging the factors connected with the milk yield of Holstein–Friesian dairy cows in Egypt. This will help researchers to understand the intricate nature of milk production in dairy cows more deeply. Furthermore, the interpretations of vital tricks and outcomes, along with the applicability of ordered logit models in animal breeding plans, have been assessed.

## Materials and methods

### Data collection, management, and mining

In this study, a total of 8300 dairy records were obtained for investigating the potential factors influencing milk production in Holstein–Friesian cows raised in Egypt. The whole records were collected from a set of commercial dairy farms, namely, Alexandria-Copenhagen and Dina farms, located about 77 km from Cairo. Materials of this investigation consisted of records of dairy cows that calved in the period from 2005 to 2019. The pedigree files represented the first six parities of lactating animals. On the farms, from time to time, all cows were kept on dirty floors, in open yards, and/or in slightly covered yards provided with cool spraying conditions during the hot weather. Animals fed on rations contained about 18% crude protein, based on the National Research Council (NRC 2001). The milking framework is automatic, in which cows are milked mechanically three times per day. The lactation records and all data are computer-based with regular updating. Data and variables used in this study were efficiently handled for the presence of outliers and missing values. According to the central limit theory, the sample size (*n* > 30) for each variable was adequate enough and proved to be sufficient for running the ordered logit models. In addition, the datasets of milk yield were normally distributed, accounting for the values of means and standard deviations of milk yield.

### Traits studied and data coding

The data included in the present research were applied to identify and model the significant factors influencing milk yield in Holstein-Friesians using the ordered logit models. Therefore, the studied variables were actual milk yield (kg), age at first calving (month), lactation order (number), days open, lactation period (days), peak milk yield (kg), and dry period (days). As shown in Table 1, considering the role of each variable in the model and how their categories have been coded, the actual milk yield was the dependent variable of interest, while other variables were fitted as the explanatory variables. To account for the potential differences among cows, the actual milk for every cow was categorized as low (< 4500 kg), medium (4500–7500 kg), and high (> 7500 kg). This categorization of milk yield was initiated on the basis of herd variations and deviations, according to previous recommendations (Akkus and Ozkoc 2012; Akkus et al. 2019; Akkus and Sevinc 2019; El-Kasrawy et al. 2020). In terms of the explanatory variables coding, age at first calving was classified into three groups (< 22 months = 1, 22–25 months = 2, and > 25 months = 3). Lactation order was categorized into six categories (from P1 to P6). Days open was divided into four classes (< 100 days = 1, 100–200 days = 2, 201–400 days = 3, and > 400 days = 4). Lactation period was divided into three categories (< 150 days = 1, 150–250 days = 2, and > 250 days = 3). Peak yield was categorized as < 35 kg = 1, 35–50 kg = 2, and > 50 kg = 3. The days’ dry variable was subdivided into three groups (< 60 days = 1, 60–80 days = 2, and > 80 days = 3). Moreover, the coding process of independent variables has been carried out based on the expert suggestion of previous authors (Topal et al. 2010; Abdallah and Abo Elfadl 2017; Akkus and Sevinc 2019; El-Kasrawy et al. 2020). Furthermore, the higher categories of such explanatory variables were proposed to be the baselines for other categories in order to understand and explain the variations in actual milk production more deeply.

**Table 1:** The variables studied and data coding

Variable	Role in the model	Categories and codes
Actual milk yield (kg)	Dependent variable (response or outcome)	< 4500 (low) 4500–7500 (medium) > 7500 (high)
Age at first calving (month)	Explanatory variable Covariate	< 22 22–25 > 25
Lactation order (parity)	Explanatory variable	P1 P2 P3 P4 P5 P6
Days open	Explanatory variable	< 100 100–200 201–400 > 400
Lactation period (days)	Explanatory variable	< 150 150–250 > 250
Peak milk yield (kg)	Explanatory variable	< 35 35–50 > 50
Dry period (days)	Explanatory variable	< 60 60–80 > 80

### The ordered logit models

In statistical modeling, identifying the correct model is the first step to be considered when the researcher is interested in developing a model with a high degree of validity and goodness. The best-suited model is mainly structured according to the nature of the dependent variable in the data. Qualitative dependent outcomes with many levels, usually with more than two categories, should be analyzed by using models presented in Table 2. To achieve this, the assumptions of each model should be taken into account so that the model can identify risk factors with a high degree of accuracy (Akkus and Ozkoc 2012). In this article, a multivariable-ordered logit model was employed to identify the risk factors affecting milk yield in dairy cows. The formula of the ordered logit model can be outlined as follows:

**Table 2:** The most common models used for modeling dependent variables with multiple levels

Model name^a^	Type of dependent variable	Assumptions
Multinomial logistic regression (logit)	Categorical, unordered	a. The characteristics of individuals should be provided b. The dependent variable categories should be independent
Multinomial logistic regression (probit)	Categorical, unordered	a. The characteristics of individuals should be provided
Ordinal logistic regression (ordered logit)	Categorical, ordered	a. The characteristics of individuals should be provided b. Proportional odds assumption is required
Ordinal logistic regression (ordered probit)	Categorical, ordered	a. The characteristics of individuals should be provided b. Proportional odds assumption is required
Nested logit	Nested nominal design	a. The inclusive value (IV) should be positive
Conditional logit	Categorical, nominal	a. The characteristics of both categories and individuals should be provided

^a^According to previous authors (Akkus and Ozkoc 2012; Akkus and Sevinc 2019)

Consider *Y* is the ordered dependent variable, and *K* + 1 is the ordered group which can be defined as follows:

1 $$\mathrm{P}\left(\mathrm{Y}\le \mathrm{j}\right)={\mathrm{P}}_{1}+\dots +{\mathrm{P}}_{\mathrm{j}}$$

The cumulative logit is incorporated to account for the ordering as follows.

Suppose that *K* + 1 is the ordered level of the variable which is stated as

2 $$\mathrm{Odds }\left(\mathrm{Y}\le \mathrm{j}\right)=\frac{\mathrm{P}\left(\mathrm{Y }\le \mathrm{ j}\right)}{1-\mathrm{ P}\left(\mathrm{Y }\le \mathrm{ j}\right)}=\frac{\mathrm{P}1 + \dots +\mathrm{ Pj}}{\mathrm{Pj }+1 \dots +\mathrm{ Pk}+1}$$

3 $$\begin{array}{cc}\mathrm{Logit}(\mathrm{Y}\le \mathrm{j})=\mathrm{Ln}(\frac{\mathrm{P}(\mathrm{Y }\le \mathrm{ j})}{1-\mathrm{ P}(\mathrm{Y }\le \mathrm{ j})}& \mathrm{j }= 1, 2, \dots ,\mathrm{ k}\end{array}$$

Then, the cumulative logit model for an ordinal dependent variable is given by

4 $$\begin{array}{cc}\mathrm{Logit}\left(\mathrm{Y}\le \mathrm{j}\right)={\mathrm{\alpha }}_{\mathrm{i}}+{\upbeta }_{\mathrm{ij}}{\mathrm{X}}_{1}+\dots \dots .+{\upbeta }_{\mathrm{ik}}{\mathrm{X}}_{\mathrm{k}}& \mathrm{j }= 1, 2, \dots \dots ,\mathrm{ k}\end{array}$$

where *Y* is the ordered variable, *α*_*i*_ is the constant term, and *β*’s are the coefficients of the logit model (estimates of the parameters for the independent variables *X*_1_, *X*_2_, ….*X*_*k*_).

The cumulative logit (odds) are consequently estimated by

5 $$\mathrm{Odds}(\mathrm{Y}\le \mathrm{j})=\mathrm{EXP}({\mathrm{\alpha }}_{\mathrm{i}})+\mathrm{EXP}({\upbeta }_{\mathrm{ij}}{\mathrm{X}}_{1}+\dots \dots . +{\upbeta }_{\mathrm{ik}}{\mathrm{X}}_{\mathrm{k}})$$

From the above equations, it could be concluded that the odds were proportional. Consequently, the proportional odds model is commonly investigated and can be written as

6 $${\mathrm{Y}}_{\mathrm{i}}={\upbeta }_{\mathrm{i}}\mathrm{X}+{\mathrm{e}}_{\mathrm{i}}$$

Because the dependent variable (*Y*) is divided into categories, we can rewrite Eq. (6) as follows:

7 $${\mathrm{K}}_{\mathrm{x}}(\mathrm{x})=\mathrm{Ln}\left[\frac{\mathrm{P}\left(\mathrm{Y }\le \mathrm{ i}\right)/\mathrm{x}}{\mathrm{P }\left(\mathrm{Y }>\mathrm{ i}\right)/\mathrm{ x}}\right]$$

Therefore,

8 $$\mathrm{Ln }(\frac{\sum \mathrm{P }(\mathrm{event})}{1-\sum \mathrm{P }(\mathrm{event})})=\mathrm{\alpha }+{\upbeta }_{1}{\mathrm{X}}_{1}+{\upbeta }_{2}{\mathrm{X}}_{2}+{\upbeta }_{3}{\mathrm{X}}_{3}+\dots +{\upbeta }_{\mathrm{k}}{\mathrm{X}}_{\mathrm{k}}$$

The formula in Eq. (8) is defined as the ordered logit (ordinal logistic) model for K independent variables and (*P*-1) subgroups of the *Y*-variable, in which *α* is the threshold parameter, *β*_1_…… *β*_*K*_ are the location parameters, and *X*_1_ to *X*_*k*_ are the risk variables. The ordered logit model can assume any type of independent data. Categorical, continuous, or ordinal data could be included in the model (Powers and Xie 2000). In terms of the assumptions required for running the OLM, the following were the most conditions that have been considered. (1) The dependent variable should be ordered categorical and easily coded. (2) OLM can assume one or many explanatory variables that could be nominal, ordinal, or continuous. (3) The multicollinearity among the IVs should be absent. (4) Proportionality of odds assumption which is the most one to be investigated and tested in OLM. The proportional odds assumption implies that every explanatory variable should have a similar effect at every cumulative odds of the ordered dependent variable (Borooah 2002). This assumption was tested using the likelihood ratio test (LRT), comparing the baseline model against the full-fitted model. A non-significance statistic reveals that the assumption of proportionality was met, and the odds ratios are interpretable.

### Threshold parameters and estimated probability

The threshold parameters and, consequently, the estimated probability for a certain level of actual milk yield were computed for the current datasets. Firstly, consider the following general linear model:

9 $$\mathrm{Y}=\sum\nolimits_{\mathrm{k}=1}^{\mathrm{k}}{\upbeta }_{\mathrm{k}}{\mathrm{X}}_{\mathrm{k }}+\mathrm{e}$$

where *β* and *e* represent the weights of the independent variables *X*’s and residuals, respectively. For the studied OLM, there was a specific order among the categories of the dependent variable. Suppose that the outcome variable has ordered levels *J*; then, the link function between these levels and the coefficients can be denoted by the following equations:

$$\begin{array}{lll}y_i=1,&Y\leq\mu_1&\\y_i=2,&\mu_1\leq Y\leq\mu_2&\\y_i=3,&\mu_2\leq Y\leq\mu_3&\\y_i=j,&\mu_{j-1}\leq Y,&\mathrm{where}\;\lbrack i=1,2,3,\dots,N\rbrack\end{array}$$

where *µ* constitutes the threshold parameter which discriminates the latent ordered structure. The estimated probability for the OLM for a given dependent level based on the independent variables could be stated as follows:

10 $$\mathrm{P}(\mathrm{y}=\mathrm{j}/{\mathrm{X}}_{\mathrm{k}})=\mathrm{F}({\upmu }_{\mathrm{j}}-\sum\nolimits_{\mathrm{k}=1}^{\mathrm{k}}{\upbeta }_{\mathrm{k}}{\mathrm{X}}_{\mathrm{k }})-\mathrm{F}({\upmu }_{\mathrm{j}-1}-\sum\nolimits_{\mathrm{k}=1}^{\mathrm{k}}{\upbeta }_{\mathrm{k}}{\mathrm{X}}_{\mathrm{k }})$$

11 $$\mathrm{P}(\mathrm{y}\le \mathrm{j})=\mathrm{P}(\mathrm{Y}\le {\upmu }_{\mathrm{j}})=\frac{\mathrm{EXP }(\mathrm{\mu j }- \sum_{\mathrm{k}=1}^{\mathrm{k}}{\upbeta }_{\mathrm{k}}{\mathrm{X}}_{\mathrm{k }})}{1+\mathrm{EXP }(\mathrm{\mu j }- \sum_{\mathrm{k}=1}^{\mathrm{k}}{\upbeta }_{\mathrm{k}}{\mathrm{X}}_{\mathrm{k }})}$$

Therefore, the estimated probabilities for the series of dependent variable categories can be given by Eqs. (12), (13), and 14 (Borooah 2002; Powers and Xie 2000; Akkus and Ozkoc 2018):

12 $$\mathrm{P}(\mathrm{y}=1)=\Omega ({\upmu }_{1}-\sum\nolimits_{\mathrm{k}=1}^{\mathrm{k}}{\upbeta }_{\mathrm{k}}{\mathrm{X}}_{\mathrm{k }})=\frac{\mathrm{EXP }(\upmu 1 - \sum_{\mathrm{k}=1}^{\mathrm{k}}{\upbeta }_{\mathrm{k}}{\mathrm{X}}_{\mathrm{k }}) }{1+\mathrm{EXP }(\upmu 1 - \sum_{\mathrm{k}=1}^{\mathrm{k}}{\upbeta }_{\mathrm{k}}{\mathrm{X}}_{\mathrm{k }})}$$

13 $$\begin{array}{c}\mathrm{P}(\mathrm{y}=2)=\Omega ({\upmu }_{2}-\sum_{\mathrm{k}=1}^{\mathrm{k}}{\upbeta }_{\mathrm{k}}{\mathrm{X}}_{\mathrm{k }})-\Omega ({\upmu }_{1}-\sum_{\mathrm{k}=1}^{\mathrm{k}}{\upbeta }_{\mathrm{k}}{\mathrm{X}}_{\mathrm{k }})\\ =[\frac{EXP (\mu 2 - \sum_{k=1}^{k}{\beta }_{k}{X}_{k }) }{1+EXP (\mu 2 - \sum_{k=1}^{k}{\beta }_{k}{X}_{k }) }]-[\frac{EXP (\mu 1 - \sum_{k=1}^{k}{\beta }_{k}{X}_{k }) }{1+EXP (\mu 1 - \sum_{k=1}^{k}{\beta }_{k}{X}_{k }) }]\end{array}$$

Thus, *P*(*y* = *j*) = 1 − Ω (*µ*_*J*−1_ $$- \sum_{k=1}^{k}{\beta }_{k}{X}_{k}$$)

14 $$=1-\left[\frac{\mathrm{EXP }(\mathrm{\mu j}-1 - \sum_{\mathrm{k}=1}^{\mathrm{k}}{\upbeta }_{\mathrm{k}}{\mathrm{X}}_{\mathrm{k }}) }{1+\mathrm{EXP }(\mathrm{\mu j}-1 - \sum_{\mathrm{k}=1}^{\mathrm{k}}{\upbeta }_{\mathrm{k}}{\mathrm{X}}_{\mathrm{k }})}\right]$$

The number of threshold parameters is equal to the number of levels of the dependent variable minus one (*P* − 1). In addition, the threshold parameters were examined for their significance. The proved significance indicates that the model *Y*-variable had an ordered structure.

### Odds ratio and goodness-of-fit tests

In order to interpret the ordered logistic regression, the Wald statistic was computed to determine the significant independent variables (IV) influencing the milk yield. For a given predictor and its levels, a *P* value ≤ 0.05 supports the significance of those factors. To quantify the contribution of each IV explaining the milk yield, the odds ratio (OR) was estimated via the program syntax and command. OR is the probability of success (P) divided by the probability of nonoccurrence (1-P). Practically, OR is the exponent of the coefficient of each explanatory variable, which can be symbolized as exp (*β*) or (*e*^*β*^). Technically, each independent variable in the datasets was divided into subclasses called categories. A certain category was fitted as a reference or baseline level, in which the OR was equal to one. Therefore, other categories of each independent variable were compared to the reference group of that variable. The computed OR that is greater than one indicates a positive association, while OR < one implies a negative association.

The quality and goodness-of-fit of OLM models in the present study were assessed using *R*-squared-based measures such as Cox and Snell R^2^, McFadden estimates, as well as Nagelkerke *R*^2^. These estimates measure the amount of variation in percentage in the ordered dependent variable accounted for by the explanatory variables (Field 2005). Additionally, the overall goodness-of-fit of the OLM was outlined using the Hosmer-Lemshow test (Hosmer and Lemshow 2002). The test is chi-square distributed with Q-2 degrees of freedom, where Q is the group interval in the dataset. A non-significant *P* value (*P* > 0.05) indicates that the OLM performs well and could be utilized for predictive purposes. Among the important model fitting information, the likelihood ratio test (LRT) was applied to ascertain how well the OLM fit to the current milk yield datasets. The LRT is basically built on the –2 log-likelihood criterion. This -2LL value is a monitor for the amount of residuals that existed in the OLM. Small values of -2LL, along with significant *P* values (*P* < 0.05), indicate a good fitting OLM. Moreover, the -2LL estimate is called the deviance because it compares the base model, the model with only intercept, against the full model (the OLM with all explanatory variables). The LRT is also chi-square distributed (with DF = *K* of base model –*K* of the full model, where *K* is the estimated parameters in the OLM) and can be formulated as follows:

$$\upchi 2=[-2\mathrm{LL}(\mathrm{base\; model})]-[-2\mathrm{LL }(\mathrm{full\; model})]$$

In addition, the OLM was assessed in terms of its validity for classification of the current dataset’s observations and further prediction of new cases using the percentage of correct classification, shortly named the classification rate. The percent of correctly classified cases was estimated through generating the confusion matrix using SPSS and Minitab statistical packages.

### Running the OLM and statistical analysis

The datasets of this study were analyzed using different statistical packages, such as SPSS, Minitab, and R. By estimating OLM, the effects of age at first calving, lactation order, days open, lactation period, peak milk yield, and dry period on the actual milk yield were investigated. The multivariable OLM was carried out using the PLUM approach (polytomous logit universal model) in the SPSS program (version 25 for windows). Other estimates and codes were obtained through Minitab and R packages. The analytical procedures were summarized as follows: (a) conducting the output management system (OMS), (b) running the PLUM steps with SPSS syntax for categorical explanatory variables that have more than two categories, (c) getting the parameter estimates of PLUM via OMS, and (d) generating the odds ratio and their corresponding confidence intervals using SPSS commands. By demonstrating these steps, the maximum likelihood estimates of the base model, Wald statistics, and odds ratio were denoted. The OLM is based on the maximum likelihood estimation using the link function. The higher categories for all variables were fitted to be the reference group. The following codes were carried out to run the ordered logit significance tests for categorical explanatory variables with more than two choices:

- DATASET ACTIVATE DataSet1.
- PLUM ACTUAL_MILK BY Lactation_order DAYS_OPEN LACTATION_PERIOD PEAK_MILK_KG AGE_FIRST_CALV.
-     DRY_PERIOD
-     /CRITERIA = CIN(95) DELTA(0) LCONVERGE(0) MXITER(100) MXSTEP(5) PCONVERGE(1.0E-6) SINGULAR(1.0E-8).
-     /LINK = LOGIT.
-    /PRINT = CELLINFO FIT PARAMETER SUMMARY TPARALLEL.
-    /SAVE = ESTPROB PREDCAT PCPROB ACPROB.
-    /TEST = Lactation_order 1 0 -1;
-               Lactation_order 0 1 -1.
-    /TEST = DAYS_OPEN 1 0 0 -1;
-               DAYS_OPEN 0 1 0 -1;
-               DAYS_OPEN 0 0 1 -1
-     /TEST = LACTATION_PERIOD 1 0 -1;
-                LACTATION_PERIOD 0 1 -1
-     /TEST = PEAK_MILK_KG 1 0 -1;
-                PEAK_MILK_KG 0 1 -1
-     /TEST = AGE_FIRST_CALV 1 0 -1;
-                AGE_FIRST_CALV 0 1 -1
-     /TEST = DRY_PERIOD 1 0 -1;
-               DRY_PERIOD 0 1 -1.

## Results and discussion

Table 3 shows the summary statistics and the dataset description for the studied variables. By considering the categories of cow’s milk yield, the average yields were 2954.80 ± 1048.47 kg, 5963.51 ± 881.89 kg, and 11,666.56 ± 3870.11 kg for the low-, medium-, and high-producing cows, respectively. The percentages, averages, and standard deviations of the explanatory variables being investigated, along with the corresponding variable levels, are presented in Table 3. For AFC, the means of the three categories were 21.86 months, 23.56 months, and 28.23 months, respectively. The lowest average of DO was estimated to be 76.81 days, while the highest was computed to be 492.26 days. The average lactation periods in days were 120.12, 200.71, and 399.01 for the three levels of LP. In terms of peak milk yield per kg, the mean values for the categories analyzed were 31.14 kg, 43.50 kg, and 56.15 kg, respectively. The dry period lengths were 55.29 days, 65.54 days, and 103.90 days on average. In general, the overall means of the examined traits were within the normal limits of Egyptian dairy herds and came close to the estimates denoted by previous investigations such as Ali et al. (2005); Abdallah and Abo-Elfadl (2017); Moawed and Osman (2018), Abd-El Hamed and Kamel (2021), and Moawed et al. (2021), who conducted multiple studies on Holstein–Friesian dairy cows raised in Egypt using different logistic regression methods. When it comes to the proportions of milk yield categories, the pooled data (Table 3) revealed that 22.72% of cows yielded low milk production (< 4500 kg), while 28.55% of cows were of medium yield (4500–7500 kg). Furthermore, 48.73% of the current hard were highly producing cows (> 7500 kg).

**Table 3:** Summary statistics and descriptive measures for the lifetime investigated variables

Dependent and independent variables	Summary Statistics		
	Percent (%)	Mean	Standard deviation
Actual milk yield (kg)			
< 4500	22.72	2954.80	1048.47
4500–7500	28.55	5963.51	881.89
> 7500	48.73	11,666.56	3870.11
Age at first calving (month)			
< 22	15.40	21.86	0.34
22–25	71.75	23.56	0.74
> 25	12.86	28.23	3.19
Days open			
< 100	32.14	76.81	12.84
100–200	37.97	144.79	27.78
201–400	20.33	284.57	54.46
> 400	7.47	492.26	88.86
Lactation period (days)			
< 150	19.43	120.12	25.37
150–250	37.52	200.71	28.31
> 250	40.96	399.01	127.29
Peak milk yield (kg)			
< 35	8.97	31.14	4.57
35–50	60.24	43.50	3.86
> 50	30.79	56.15	4.03
Dry period (days)			
< 60	49.78	55.29	3.80
60–80	40.36	65.54	4.61
> 80	9.72	103.90	26.74

The results of OLM estimating and predicting the determinants influencing the actual milk yield of the Holstein–Friesian cows are summarized in Tables 4, 5, and 6. This OLM was estimated using the maximum likelihood methodology. The results of the likelihood ratio test (Table 4) revealed that the assumption of proportional odds was met. The chi-square statistics (23.84) was statistically non-significant (*P* value > 0.05; at df = 16), suggesting the acceptance of the null hypothesis which supposes that the coefficients of explanatory variables are constant all over the categories of milk yield. Based on the results of LRT, it can be concluded that the OLM is proportional, both in the log-odds and odds ratio. A study carried out by Adeleke and Adepoju (2010), who estimated a similar model, showed that the non-significance result of LRT indicates that the lines of slopes are parallel throughout the levels of the response variable, denoting what is called the assumption of parallelism. The present finding of LRT that was created on the logit link function is in accordance with previous studies (Hosmer and Lemeshow 2000; Dohoo et al. 2009; Agga and Scott 2015) which used the Brant test to examine the proportionality assumption. These results support the validity of our OLM to the milk yield datasets, and consequently, the coefficients and odds ratio could be easily and perfectly interpreted.

**Table 4:** Test of parallel lines (proportional odds assumption)

Tested model	-2 log-likelihood^*^	Chi-square	Degrees of freedom (df)	*P*-value
Null hypothesis	374.9			
General	351.1	23.84	16	0.093^NS^

NS: non-significant at 5% significance level (*P* > 0.05)

**Table 5:** Model fitting, validity, and goodness-of-fit information based on the logit link function

Model fitting information				
Model	-2 log-likelihood	Chi-square	DF	*P* value
Intercept only	1213.7			
Final	374.9	838.8	16	0.0001*
Goodness-of-fit measures				
		Chi-square	DF	
Pearson		378.4	598	1.00
Deviance		286.5	598	1.00
Pseudo *R*-square estimates				
Cox and Snell			0.722	
Nagelkerke			0.823	
McFadden			0.609

**Table 6:** Multivariable-ordered logit model for the analysis of factors influencing milk yield of Holstein-Friesians

Dependent variable: actual milk yield 1: < 4500 2: 4500–7500 3: > 7500	Coefficients: β (SE)	Wald	*P* value	95% CI of coefficients		Odds ratio (OR)
				Lower limit	Upper limit	
Threshold parameters						
Actual milk yield (1) *µ*_1_ = − 26.936	0.948	806.6	0.0001^**^	− 28.79	− 25.08	-
Actual milk yield (2) *µ*_2_ = − 22.652	0.834	737.8	0.0001^**^	− 24.29	− 21.02	-
Location parameters						
Age at first calving						
< 22	0.730 (0.456)	2.567	0.039^*^	− 1.163	1.623	2.08
22–25	0.358 (0.367)	0.948	0.330^NS^	− 0.362	1.077	1.43
> 25 (base group)						
Days open						
< 100	− 18.54 (0.391)	2247.9	0.001^**^	− 19.31	− 17.78	0.432
100–200	− 19.01 (0.373)	2602.4	0.001^**^	− 19.75	− 18.28	0.369
201–400	− 17.76 (0.965)	338.56	0.048^*^	− 19.26	− 16.26	0.721
> 400 (base group)						
Lactation period (DIM)						
< 150	− 9.78 (0.654)	224.19	0.001^**^	− 11.07	− 8.51	0.509
150–250	− 4.71 (0.393)	143.62	0.001^**^	− 5.48	− 3.94	0.489
> 250 (base group)						
Peak milk yield						
< 35	− 5.39 (0.512)	110.98	0.001^**^	− 6.39	− 4.39	0.691
35–50	− 2.58 (0.349)	54.71	0.042^*^	− 3.26	− 1.89	0.856
> 50 (reference)						
Dry period						
< 60	0.406 (0.448)	0.823	0.044^*^	− 0.471	1.284	1.50
60–80	− 0.031 (0.449)	0.005	0.945^NS^	− 0.912	0.850	0.97
> 80 (base group)						
Lactation order (parity)						
P1	0.255 (0.517)	0.242	0.623^NS^	− 0.759	1.268	1.29
P2	0.599 (0.518)	1.341	0.047^*^	− 0.415	1.614	1.82
P3	0.045 (0.533)	0.007	0.933^NS^	− 1.00	1.090	1.05
P4	0.553 (0.575)	0.924	0.036^*^	− 0.574	1.679	1.74
P5	0.148 (0.656)	0.051	0.821^NS^	− 1.137	1.433	1.16
P6 (base group)						
Model validity						
-2 log-likelihood = 374.9; LR chi-sq (16) = ; prob > chi-square = 0.0001^**^						
Correct classification rate = 82.32%						

^**^Coefficient is significant at a 0.01 level of significance (*P* < 0.01)

^*^Coefficient is significant at a 0.05 level of significance (*P* < 0.05)

^NS^Coefficient is non-significant at a 0.05 level of significance (*P* > 0.05)

Reference categories: AFC (> 25 months), DO (> 400 days), LP (> 250 days), PMY (> 50 kg), DP (> 80 days), lactation order or parity (P6)

The model fitting information and goodness-of-fit diagnostic measures for the estimated OL model are shown in Table 5. The first information to be outlined was the result of LRT that has been used to assess the differences between the -2 log-likelihood of the intercept-only model against the full model. This difference was demonstrated by the chi-square value (838.8) which appeared to be highly significant (*P* < 0.001, df = 16), indicating that the present OLM with full information is statistically fit to data better than using the model with only constant term (Manu et al. 2020). Moreover, the Pearson chi-square statistics (378.4) was statistically insignificant at 0.05 level of significance (*P* > 0.05) with df = 598. This statistic showed that the overall OL model is fit. Similarly, the deviance chi-square value (286.5, *P* > 0.05) confirms the overall goodness-of-fit of the OLM to the milk production dataset. In conclusion, similar to the previous studies (Ali et al. 2005; Adeleke and Adepoju 2010; Abdallah and Abo-Elfadl 2017; Manu et al. 2020), the present results showed the applicability of OLM we have applied for analyzing dairy records. In terms of the ability of OLM to explain the variability among milk yield categories, the pseudo-*R*-square estimates (Cox and Snell = 0.722, Nagelkerke = 0.823, and McFadden = 0.609) indicate that at least 61.0% of the variation in the likelihood of denoting a high level of milk yield were explained by the chosen independent variables. It can be noticed that the present findings of model efficiency estimates (Tables 4 and 5) were higher than those reported by previous studies (Abdallah and Abo-Elfadl 2017; Akkus and Sevinc 2019; Akkus et al. 2019) who applied the ordinal logistic regression models to assess and predict milk yield and time series data of Holstein–Friesian cows.

The multivariable adjusted-ordered logit model results involving the estimated coefficients of the location parameters testing the significance of variable levels, their standard errors, Wald statistics, probability values, as well as the odds ratios are given in Table 6. When this table is evaluated, it can be obviously observed that the threshold parameters (*µ*_1_ =  − 26.936 and *µ*_2_ =  − 22.652) were statistically significant (*P* < 0.001), as denoted by the Wald statistics. The significant results of threshold parameters imply that the actual milk yield as a dependent variable is an ordinal variable, and therefore, the reliability of the OLM to the current datasets was verified as hypothesized prior to statistical analysis. This finding is in accordance with those reported by Moawed and Osman (2018), Akkus et al. (2019), Akkus and Sevinc (2019), and Manu et al. (2020). In terms of the model validity, the value of the -2 log-likelihood of the likelihood ratio test was highly significant (-2LL = 374.9, chi-square at df = 16, *P* < 0.001). This result is another evidence of the suitability of the OLM to the data structure. Additionally, the percent of cases correctly classified by the OLM was 82.32%. For every explanatory variable besides its examined categories, the estimated coefficients (with their standard errors) are presented in Table 6. The Wald statistics and *P*-values are also demonstrated for testing the significance of the parameters estimated by the model. Overall, a negative coefficient means that the corresponding factor influences the actual milk yield in a decreasing manner, while a positive coefficient indicates that the factor level increases milk yield with regard to the reference category (Akkus et al. 2019). When age at first calving is examined, it was noticed that cows with AFC < 22 months had a statistically significant (*P* < 0.05) impact on milk yield compared to older cows. In other words, younger cows appear to positively (*β* = 0.730) and statistically (*P* = 0.039 < 0.05) produce a higher amount of milk yield. The odds ratios are interpreted only for the statistically significant factor levels. The OR for cows with AFC < 22 months was estimated to be 2.08. When this value is interpreted close to the base group (cows with AFC > 25 months, Table 6), it can be concluded that the probability that a cow will yield a higher amount of milk (greater than 4500 kg) will be two times higher for those with AFC < 22 months.

It can be concluded that cows calving for the first time at young ages (< 22 months) produce the highest amount of milk yield compared to cows with older ages (> 25 months). This result agrees with those reported by Akkus et al. (2019) who conducted a similar study using OLM to outline factors influencing milk yield in Holstein-Friesians. The present finding is also supported by the outcome of Eastham et al. (2018) and Nilforooshan and Edriss (2004) who reported that lower ages at first calving lead to higher quantities of milk yield. This may occur because cows with high AFC could be exposed to nutritional and reproductive disorders that delay fertility and subsequently reflect on milk production traits (Kocak and Ekiz 2006; Aytekinrural et al. 2016).

According to the model estimated (Table 6), it was noticed that shorter and moderate days open caused a significant (*P* < 0.05) decrease in milk yield compared to longer DO (> 400 days) because of the negative coefficients estimated by the model. In other words, cows having longer DO yielded milk more than those with shorter and moderate calving-conception intervals. The ORs for all categories of DO were less than one; hence, these values were inverted to denote more interpretable conclusions (El-Awady et al. 2021; Habibi et al. 2021). This means, compared to DO < 100 days, the probability that a cow will yield milk of more than 4500 kg will be 2.31 (1/0.432) times greater for those of DO > 400 days. Similarly, compared to DO between 100 and 200 days, the likelihood of cows that will give milk amounts more than 4500 kg will be 2.71(1/0.369) times higher for those of DO > 400 days. The OR for DO between 201 and 400 days was 0.721. The inverse of this value is 1.39, suggesting that the probability of milk production is 1.39 times higher for cows having DO > 400 days.

The results showed that lactation length has a significant impact (*P* < 0.01) on the milk yield at the two examined levels. The estimated coefficients (Table 6) revealed that milk yield was fluctuated according to the lactation period. Compared to the reference category (LL > 250 days), the other levels (LL < 150 days and LL from 150–250 days) showed a statistically significant decrease in milk production due to the negative signs of the estimated coefficients. This means that the higher the LL, the higher the amount of milk produced by cows. The odds ratios for the two levels of LL were 0.509 and 0.489, respectively, which yields nearly 2.0 when inverted. Subsequently, the probability that cows will yield high milk quantities is two times higher for longer LL (> 250 days). This result is in agreement with the outcomes of Vijayakumar et al. (2017) and Akkus et al. (2019) who reported that Holstein cows denote a higher amount of milk in longer lactation lengths. Moreover, Lateef et al. (2008) mentioned that the maximum amount of milk is gained from HF cows having the highest lactation periods. Additionally, the peak yield per kg has a significant effect on the milk yield (*P* < 0.05). The odds ratios for the two levels were less than one, meaning that the estimated coefficients were negative (− 5.39 and − 2.58). The inverse of these ORs were 1.45 and 1.17 for the peak yield levels; < 35 kg and 35 to 50 kg, meaning that the probability of milk production will be 1.45 times higher for cows having a peak yield more than 50 kg as compared to those showing peak yield less than 35 kg.

One of the most economic and functional traits studied was the dry period impact. The present result (Table 6) revealed that a shorter dry period (< 60 days) was statistically associated with milk yield at a probability level of < 0.05. The odds ratio of the shortest DP category (< 60 days) was 1.50. When comparing this value with the base category (DP > 80 days), it could be concluded that the probability that cows may produce milk higher than 4500 kg will be 1.5 times higher for the shorter DP. On the other hand, DP length of 60 to 80 days has no significant (*P* > 0.05) effect on milk yield as compared to DP greater than 80 days. This result was confirmed by the value of OR which appeared to be close to one. The present result comes in accordance with the findings of previous authors (Mansfeld et al. 2012; Kok et al. 2017, 2021; Boustan et al. 2021) who conducted several studies connected to the effect of DP on milk yield. In conclusion, their results showed that shortening the length of DP (≤ 60 days) leads to an improvement in the metabolic status of dairy cows without any decrease in total milk production.

When the parity is assessed, results showed that milk yield was significantly affected by both the 2nd and 4th parities, as given in Table 6. For the 2nd parity, the model coefficient (0.599) was positive and statistically influenced the milk yield with a *p*-value of 0.047. Additionally, the 4th parity denoted similar results to the 2nd parity in regard to the estimated coefficient and its significance. Putting the results together, it can be concluded that milk yield was increased in the two parity levels compared to other parities. The odds ratios of the 2nd and 4th parities were 1.82 and 1.74, respectively. Hence, the probability that cows will produce milk amounts higher than 4500 kg is 1.82 and 1.74 times higher for the 2nd and 4th parities, respectively, as compared to the 6th parity, the base level. Previous studies that investigated OLM, such as the study of Akkus and Sevinc (2019), reported that the 4th parity level significantly affected milk yield with an estimated coefficient equal to 0.406. On the other hand, M’hamdi et al. (2012) and Akkus et al. (2019) showed that cows on the 1st parity denoted less milk compared to cows on its 6th lactation order.

The predicted OLM and, consequently, the estimated probabilities can be easily provided for the three levels of actual milk yield based on the characteristics of independent variables of the model. The right side of the OL model can be computed by using the available information of independent variables for every cow in the herd as follows:

$$\begin{array}{l}\sum\nolimits_{k=1}^k\beta_kX_k=\lbrack+0.730\mathrm{AFC}(<22)+0.358\mathrm{AFC}(22-25)-18.54\mathrm{DO}(<100)-\\19.01\mathrm{DO}(100-200)-17.76\mathrm{DO}(201-400)-9.78\mathrm{LP}(<150)-\\4.71\mathrm{LP}(150-250)-5.39\mathrm{PMY}(<35)-2.58\mathrm{PMY}(35-50)+0.406\mathrm{DP}(<60)-\\0.031\mathrm{DP}(60-80)+0.255\mathrm P1+0.599\mathrm P2+0.045\mathrm P3+0.553\mathrm P4+0.148\mathrm P5\rbrack\end{array}$$

To consider the estimated probability for each category of milk yield separately, the following procedures were demonstrated as follows.

Based on the contained OLM, the estimated probability that the actual milk yield of cows will be < 4500 kg given that the AFC < 22 months, DO < 100 days, LP between 150 and 250 days, PMY between 35 and 50 kg, and DP < 60 days, and for the second parity (P2), holding the other factor levels were fixed was calculated as follows:

$$\sum\nolimits_{k=1}^{k}{\beta }_{k}{X}_{k }=[+0.730 (1) - 18.54 (1) - 4.71 (1)- 2.58 (1) + 0.406 (1) + 0.599 (1)]$$

For low milk yield, *P*(*y* = 1) = $\frac{EXP [\left(-26.936 - \sum_{k=1}^{k}{\beta }_{k}{X}_{k }\right)] }{1+EXP [\left(-26.936 - \sum_{k=1}^{k}{\beta }_{k}{X}_{k }\right)]}$

$$=\mathrm{P}(\mathrm{y}=1)=\frac{\mathrm{EXP }[(-26.936 -\left(-24.095\right)] }{1+\mathrm{EXP }[(-26.936 -\left(-24.095\right)]}$$

$$=\mathrm{P}(\mathrm{y}=1)=\frac{\mathrm{EXP }[-2.841] }{1+\mathrm{EXP }[-2.841]}$$

$$=\mathrm{P}(\mathrm{y}=1)= \frac{0.058367 }{1.058367}$$

The estimated probability = 0.055148.

By the way, the probability that the milk yield will be between 4500 and 7500 kg for the same conditions was estimated by the following:

For middle milk yield, *P*(*y* = 2) = [$\frac{EXP (\mu 2 - \sum_{k=1}^{k}{\beta }_{k}{X}_{k }) }{1+EXP (\mu 2 - \sum_{k=1}^{k}{\beta }_{k}{X}_{k })}$] – [$\frac{EXP (\mu 1 - \sum_{k=1}^{k}{\beta }_{k}{X}_{k }) }{1+EXP (\mu 1 - \sum_{k=1}^{k}{\beta }_{k}{X}_{k })}$]

$$=[\frac{EXP (-22.652 - \sum_{k=1}^{k}{\beta }_{k}{X}_{k }) }{1+EXP (-22.652 - \sum_{k=1}^{k}{\beta }_{k}{X}_{k }) }]-[\frac{EXP (-26.936 - \sum_{k=1}^{k}{\beta }_{k}{X}_{k }) }{1+EXP (-26.936 - \sum_{k=1}^{k}{\beta }_{k}{X}_{k }) }]$$

$$=[\frac{EXP (1.443) }{1+EXP (1.443) }]-[\frac{EXP (-2.841) }{1+EXP (-2.841) }]$$

$$=[\frac{4.23337 }{5.23337 }]-[\frac{0.05837 }{1.05837 }]$$

$$=0.80892-0.055148$$

The estimated probability = 0.75377.

Consequently, the probability that the milk yield will be between above 7500 kg using the recommended OLM was found to be.

For high milk yield, *P* (*y* = 3) = 1 – [$\frac{EXP (\mu j-1 - \sum_{k=1}^{k}{\beta }_{k}{X}_{k }) }{1+EXP (\mu j-1 - \sum_{k=1}^{k}{\beta }_{k}{X}_{k })}$]

$$=1-[\frac{\mathrm{EXP }(-22.652-(-24.095) }{1+\mathrm{EXP }(-22.652-(-24.095)}$$

The estimated probability = 0.19108.

It was noticed that the sum of the estimated probabilities (0.055148 + 0.75377 + 0.19108) denoted at the three ordered categories of the milk yield was equal to one.

## Conclusion

The present study was designed to determine the most important factors influencing the actual milk yield of Holstein–Friesian cows using the ordered logit model. Unlike the other classical regression models, the OLM was proven to be superior and advantageous when dealing with the ordinal outcome. The main benefit of the OLM is that it can address the significant contribution of each level of the dependent variable through interpreting the odds ratios against the reference categories. Moreover, the direction of the relationship between the dependent variable levels and the predictors was computed, enabling us to identify the levels connected with a positive and negative change in milk production. Additionally, the estimated probabilities are also perfectly provided for each level of the three groups of milk yield based on the investigated characteristics. In conclusion, the ordered logit modeling can be utilized to analyze livestock datasets, allowing researchers to find out the most significant factors connected with milk yield as an economic characteristic of farm animals. This could allow the farm owners to plan for the necessary improvements in the future. Using this technique, the significance of the factors influencing the actual milk yield in dairy cows, such as age at first calving, lactation order, days open, lactation period, dry period, and peak milk yield, was identified, considering the different levels of each factor. In the final model, it was noticed that the fluctuation in milk yield did not only depend on the whole factor, but it was also more affected by its levels, showing a significant change in milk yield over time via the odds ratio estimated. To date, a limited number of studies have been designed to apply the OLM in the field of animal production. Therefore, by considering the present findings and the advantages provided by this statistical methodology, it could be used in the future in promising studies for perfect improvement of the milk yield connected traits. The breeding strategies should consider the significant factors investigated. Similar research could be designed by incorporating more number of cows and more variables.

## Data Availability

The datasets analyzed during the current study are available from the corresponding author upon request.

## Code availability

SPSS version 25/Minitab version 15/R version 4.1.2.
